# Evaluating the Chemical Components and Flavor Characteristics Responsible for Triggering the Perception of “Beer Flavor” in Non-Alcoholic Beer

**DOI:** 10.3390/foods9121914

**Published:** 2020-12-21

**Authors:** Scott Lafontaine, Kay Senn, Laura Knoke, Christian Schubert, Johanna Dennenlöhr, Jörg Maxminer, Annegret Cantu, Nils Rettberg, Hildegarde Heymann

**Affiliations:** 1 Department of Viticulture and Enology, University of California Davis, 595 Hilgard Lane, Davis, CA 95616, USA; acantu@ucdavis.edu (A.C.); hheymann@ucdavis.edu (H.H.); 2 Department of Food Science and Technology, University of California Davis, 392 Old Davis Rd, Davis, CA 95616, USA; ksenn@ucdavis.edu; 3 Versuchs- und Lehranstalt für Brauerei in Berlin (VLB) e.V., Seestrasse 13, 13353 Berlin, Germany; l.knoke@vlb-berlin.org (L.K.); c.schubert@vlb-berlin.org (C.S.); j.dennenloehr@vlb-berlin.org (J.D.); j.maxminer@gmx.de (J.M.); n.rettberg@vlb-berlin.org (N.R.)

**Keywords:** non-alcoholic beer (NAB), sensory and chemical analyses, consumer science, beer flavor

## Abstract

Forty-two commercial non-alcoholic beer (NAB) brands were analyzed using sensory and chemical techniques to understand which analytes and/or flavors were most responsible for invoking the perception of “beer flavor” (for Northern Californian consumers). The aroma and taste profiles of the commercial NABs, a commercial soda, and a carbonated seltzer water (n = 44) were characterized using replicated descriptive and CATA analyses performed by a trained sensory panel (i.e., 11 panelists). A number of non-volatile and volatile techniques were then used to chemically deconstruct the products. Consumer analysis (i.e., 129 Northern Californian consumers) was also used to evaluate a selection of these NABs (i.e., 12) and how similar they thought the aroma, taste and mouthfeels of these products were to beer, soda, and water. The results show that certain constituents drive the aroma and taste profiles which are responsible for invoking beer perception for these North American consumers. Further, beer likeness might not be a driver of preference in this diverse beverage class for Northern Californian consumers. These are important insights for brewers planning to create products for similar markets and/or more broadly for companies interested in designing other functional/alternative food and beverage products.

## 1. Introduction

There is evidence that non-drinkers, Millennials, and Generation Z consumers are driving increased demand for healthier beverage alternatives to alcohol [1]. Therefore, the global NAB market is projected to significantly expand and multi-national brewing companies and craft breweries are beginning to prioritize the development of non-alcoholic, low-alcohol, and alcohol-free brands to meet these demands. Historically and currently, lager-type/low-calorie beer styles have been and are still the core beer styles produced and globally distributed by major multi-national brewing companies [2]. Thus, it is not surprising that a majority of non-alcoholic brands have been designed to have some of the main characteristics of these flagship alcoholic lager brands [3]. Likewise, most past research investigating beer flavor has also been focused on lager beer flavor and/or stability [4]. Therefore, lager styles tend to frame the general discussion of what brewers and/or researchers consider “beer flavor”.

However, when compared to other countries, such as Germany, legally the U.S. has a much less rigid formal definition involving the ingredients from which beer can be constructed [5]. This is evident by the increased consumer popularity and production of craft brands, which has resulted in a significant variety of beer styles to be commercially produced. In 2020, the Brewers Association recognized over 140 substyles of beer which were generally split into three main production techniques/styles—ales (78 styles), lagers (33 styles), and hybrid/mixed lagers or ales (34 styles) [6]. However, not only are there a wide variety of styles being produced, but there is also clear evidence that some of these styles now comprise a significant amount of market share and have had a profound impact on the industry, specifically brands with unique hop-forward aroma profiles along with low to moderate bitterness (such as India Pale Ales (IPAs)) [7]. The success of these products is likely based on the fact that their flavor is more complex and intense as compared to lager beer and because of this they not only attract classical beer drinkers but also introduce new consumers to beer. Recent research has also indicated that some of the most preferred commercial NABs were those with citrusy, tropical, stone fruit aromas driven by volatiles extracted from botanicals (i.e., citrus juice or dry hopping) [8]. Therefore, the aroma, taste and mouthfeel profiles of these styles might also be important considerations for brewers and researchers in the development of novel beer/adult alternative beverages. While there have been numerous studies to investigate what non-volatile and volatile constituents influence specific aroma, tastes, and mouthfeels [9,10,11,12], there has been no prior research to investigate which of these factors trigger consumers to perceive a product as more “beer like” and/or whether consumers prefer NABs which are more “beer like”.

For example, from a taste perspective, bitterness is a defining characteristic among many of these different beer styles. Recently, bitterness was also identified as a key characteristic influencing the consumer preference towards different styles of NABs and consumers were least satisfied with very bitter NABs [8]. While residual extract (i.e., sweetness) can act to suppress and balance bitterness, the constituents mainly responsible for driving changes in beer bitterness are derived from hops and different hopping techniques (i.e., iso-α-acids and humulinones). Iso-α-acids, the main drivers of beer bitterness, are formed during wort boiling and kettle hopping when α-acids are extracted from hops and undergo an isomerization dependent on temperature and time [13,14]. Therefore, the length of boiling time and the amount of α-acids added during kettle hopping have a direct impact on the concentrations of iso-α-acids and ultimately the resulting beer bitterness. Further, extracts containing iso-α-acids are also sold and can be added to fine-tune bitterness in the cellar [15]. More recently, to add significant hop aroma but not hop bitterness, craft brewers have employed a technique called dry hopping. Dry hopping is a “cold” extraction of non-volatile and volatile compounds out of hops into beer, NAB, and/or water. Humulinones (i.e., oxidized α-acids) are naturally present in hops and are extracted during dry hopping [16]. They are ~2/3 as bitter as iso-α-acids and, at high dry hopping rates, have also been shown to impact the bitterness of beer [10]. Thus, while these constituents are unique to commercial beer/NAB production and there is generally a good understanding in the brewing industry of how they influence beer bitterness. It remains unclear whether iso-α-acids and humulinones trigger consumers to perceive different beer/NAB styles as more “beer like”. The same is true for constituents driving other aromas, tastes, and mouthfeels in beer.

Consequently, having a more in-depth understanding of what constituents in different NAB styles invoke beer similarity for consumers as well as whether consumers prefer these characteristics could help brewers design more preferable NABs that more successfully meet consumer expectations and allow for more accurate and effective marketing strategies for these adult alternative beverages. Therefore, the goals of this manuscript were to (1) determine whether certain aromas, tastes, and/or mouthfeels resulted in the consumer panel and the trained panelists perceiving commercial NAB products as more “beer like”, (2) evaluate the non-volatile and volatile constituents which result in the perception of “beer”, “soda”, and “water/flavor water” in these products, and (3) evaluate whether these attributes impacted the preference of consumers towards these product

## 2. Materials and Methods

A large portion of the following materials and methods section has been published previously [8] and can be accessed by the following link https://pubs.acs.org/doi/10.1021/acsomega.0c03168 in “Section 2”. Permission was granted by the American Chemical Society to republish this section in this manuscript. Any further copyright permissions questions related to the material within this section should be directed to the American Chemical Society (support@services.acs.org). However, it should be noted that all the figures and tables in this manuscript are unique to this manuscript.

### 2.1. Collection Details and Sample Handling Protocol for Non-Alcoholic Beers, Soda, and Seltzer

For sensory and chemical analyses, a soda, a seltzer, and a diverse set of 42 NABs, comprised of several different styles and multiple different production methodologies, were obtained from 6 different countries (Table 1). Due to the propensity of NAB aroma and flavor to be impacted by staling reactions, most of the 42 NABs analyzed for this study were either donated or purchased directly from breweries, shipped straight to the University of California Davis (UC Davis) at the end of August/beginning of September 2019 and then stored at −2 °C until chemical and sensory analysis. For those that were not procured directly from breweries, eleven NABs (i.e., IPA3, lager (L) L5b, L7, L11, L14, pale ale (P) P3, P6, radler (R) R1, stout (S) S1, and wheat (W) W4) were obtained from Total Wine and More (Bethesda, MD), one was purchased from Whole Foods Market (Austin, TX) (i.e., hop water (HW) HW1), and the soda as well as the seltzer were acquired from Safeway Inc. (Pleasanton, CA). Directly after purchasing, these samples were stored under the same conditions mentioned above. For each sample, the package codes were matched to ensure all of a given product originated from the same batch. All the products were <3 months old at the time of evaluation and/or were evaluated before the best buy date labeled on the package.

For consumer analysis, based on the initial sensory and chemical data, the 12 NABs with some of the most different flavor and chemical profiles were selected from the list of 42 NABs for consumer analysis (colored in light blue in Table 1; HW2, IPA1, IPA3, L5c, L6, L9, L16, L18, R1, W1, and W4). In general, although most of these samples were from different production batches, they were procured, stored, and evaluated under similar circumstances to what was mentioned above.

### 2.2. Sensory: Descriptive and Check-All-That-Apply Analyses

Descriptive and check-all-that-apply (CATA) analyses were performed based on published methodology by a trained panel to characterize the sensory profiles (i.e., aroma, taste and mouthfeel) of the 42 NABs, soda, and seltzer [17,18]. Further details on why this methodology was used to analyze the products are outlined in Lafontaine, Senn, Dennenlöhr, Schubert, Knoke, Maxminer, Cantu, Rettberg and Heymann [8]. These sensory analyses were performed in the J. Lohr wine sensory room at the UC Davis over four weeks in September/October 2019. For the panel, 11 judges (6 females and 5 males) who had self-identified that they consumed beer on a regular basis, had a high preference for beer, and were available to participate in all of the study timeslots were recruited from students, staff, and friends of UC Davis. The protocol for this study (IRB project number 1468002-1) was exempt by the internal regulatory board.

Panel participants were trained over five 60 min training sessions. Over four of these sessions the panel blindly and randomly evaluated all the samples (in sets of 11) to establish by censuses the sensory terms and corresponding reference standards which best described the differences between the samples. While the panelists could use their own vocabulary to describe the samples, to help guide term generation, panelists were also given the second edition Beer Flavor Map™ by DraughtLab (Flavor LAB, LLC). Due to the number of different NAB styles being evaluated, 41 different terms were generated to describe the aroma, taste, and mouthfeel of the samples. Therefore, to make the data collection less fatiguing on the judges, DA was only performed on terms which the panelists decided they could scale over most of the samples (i.e., 5 aroma terms and 7 taste/mouthfeel terms). For terms more unique to specific products CATA was used (i.e., 23 aroma terms and 6 taste/mouthfeel terms). Again, more specific details involving these terms have been published [8]. The final training session was used as a practice to guide the panelists through the testing environment.

Before testing, judges were instructed to smell through labeled aroma standards. Then for a warmup before each testing session, the judges had to randomly identify a selection of the aroma standards, and to prevent fatigue, only weekly, was this same exercise was performed additionally with the taste and mouthfeel standards. During testing, 60 mL of sample was evaluated at ~14.1 °C by the judges in Libbey 5 oz. Belgian beer tasting glasses covered with plastic lids and coded with randomized three-digit numbers that differed for each judge. The samples were evaluated ~30 min after opening the container and the judges assessed the samples in individual, ventilated, and light-isolating tasting booths under red light.

The testing was performed over three replications in a randomized and balanced Latin square order designed by FIZZ (version 2.47B, Biosystèmes, Couternon, France). Scaling for DA was performed on a 12.5 cm line scale anchored by the wording “less intense” to “more intense”. Judges first scaled the DA aroma terms for each beer (both ortho and retronasal). The judges were then instructed to check all the other attributes from the list of CATA aroma terms which best described the unique aroma quality of the NABs. Following this, the panelists then scaled the basic taste and mouthfeel attributes and again checked the attributes from a list of CATA mouthfeel terms that best described the NABs. Panelist responses were collected on Chromebook tablets using Qualtrics (Provo, UT, USA). Judges were required to smell the samples, then swallow the samples, and then forced to wait for 30 s between samples while rinsing with carbonated (20 psi) 0.1% pectin water to cleanse their palates. Eleven samples were evaluated in one session with a forced three-minute break after six samples. Upon the completion of the three testing replications, three additional sessions were then performed in a randomized fashion to collect hedonic data (using a 9-point hedonic scales) as well as to scale how close the judges thought the products were to beer, soda, and sparkling water/flavored water using 12.5 cm line scale in Qualtrics anchored by the wording “not similar” to “very similar”, with the middle being “neither similar nor dissimilar”. These values were then converted onto a 9-point scale. At the end of each session, judges were given food and were compensated with a gift card upon the completion of the study.

### 2.3. Sensory: Consumer Preference Analysis

As outlined in Section 2.1, based on the initial sensory and chemical data, 12 NABs with some of the most diverse chemical and flavor profiles were selected out of the list of 42 NABs for consumer analysis (colored in light blue in Table 1). Consumer analysis was performed in the J. Lohr wine sensory room at the UC Davis over twelve one-hour sessions on a Saturday and Sunday in November 2019 (i.e., 6 sessions per day) with a 30 min break between sessions to help set up for the next session. 144 Northern Californian consumers of beer and/or interested in the assessment of beer flavor were recruited from students and staff of UC Davis and/or from the Davis community (i.e., 12 consumers per session). During testing 60 mL of sample was served at ~14.1 °C to the judges in Libbey 5 oz. Belgian beer tasting glasses covered with plastic lids and coded with randomized three-digit numbers that were the same for each consumer. The samples were evaluated ~30 min after opening the container and the judges assessed the samples in individual, ventilated, and light-isolating tasting booths under red light. The serving presentation of the samples between judges was based on a randomized and balanced Latin square order designed by FIZZ (version 2.47B, Biosystèmes, Couternon, France).

The consumer testing session was broken into four general parts. First, consumers were introduced to the testing environment and were then served 6 samples to evaluate. Consumers were asked to rate (using a 9-point hedonic scale) how much they liked the overall aroma, taste, and mouthfeel of the sample and then to individually evaluate how much they liked just the aroma as well as just the taste, and mouthfeel for each sample (again using a 9-point hedonic scale). For each sample, the consumers were also given an option to freely profile what they liked or disliked about the sample, asked to rate how similar the sample was to beer, soda, and sparkling water/flavored water (using a 9-point similarity scale, again defined in Table 1), and whether or not they would purchase the sample. Consumers were then forced to wait for 30 s between the samples and instructed to rinse with carbonated (20 psi) 0.1% pectin water to cleanse their palates. The consumers were then prompted to fill out a demographics survey (i.e., 13 questions, ~2–3 min). Following this, the consumers evaluated another 6 samples in an identical way to the first set. To finish testing the consumers then filled out an additional survey (i.e., 16 questions, ~3–5 min) which gauged their general preferences around purchasing and consuming NABs. Consumer responses were collected on Chromebook tablets using Qualtrics (Provo, UT, USA). At the end of each session, the consumers were given food, compensated with a gift card, and instructed not to conversate about the study. Consumers who were under 21 did not self-identify as American consumers, and/or had not been U.S. residents for > 5 years were removed and not considered in the data analysis (i.e., resulting in final data set generated by 129 consumers—66 males and 63 females).

### 2.4. Non-Volatile and Volatile Analyses of Non-Alcoholic Beers

Simultaneous to sensory analysis and consumer analysis, several standard and published methods were used to chemically deconstruct both the non-volatile and volatile profiles of the NABs.

#### 2.4.1. Non-Volatile Beer Analyses

Most of the basic non-volatile quality specifications are reported in Table 1. These quality specifications were measured in duplicate or triplicate for the samples evaluated in both the sensory and consumer studies. The density, real extract (Er), and alcohol content by weight (ABW) of decarbonated samples were measured with a DMA 4100M and Alcolyzer Plus (Anton Paar, Ashland, VA, USA). The pH and titratable acidity (TA) were determined with Orion™ Versa Star Pro™ advance electrochemistry meter with an Orion™ ROSS Ultra™ Refillable pH/ATC Triode™ (8157BNUMD) (Thermo Fisher Scientific, Waltham, MA, USA). For the TA measurements, the standard protocol outlined by the American Society of Brewing Chemists (ASBC) was followed [19]. In brief, 0.1 M NaOH was used to titrate the decarbonated samples at 25 °C to pH 8.2, and then the titratable acidity was reported as lactic acid %.

Bitterness units (BUs) and color were determined by both ASBC and European Brewing Congress (EBC) standard protocol, respectively, using a Hach^®^ DR6000 Spectrophotometer (Hach^®^, Loveland, CO) [19]. BUs were determined by adding 10 mL of cold carbonated beer (~2 °C), 20 mL of 2,2,4-Trimethylpentane (TMP, isooctane), and 1 mL of 3 N HCl to a 50 mL centrifuge tube and shaking for 15 min with a mechanical wrist action shaker. The tubes were then centrifuged for 15 min at 5000 rpm and the absorbance of the TMP (top) layer was measured at 275 nm and multiplied by 50 to report the BU value. For the color analysis, the absorbance of decarbonated beer at 25 °C was measured at both 430 and 700 nm. To report the EBC color value, the absorbance at 430 nm was multiplied by 25. Samples with excessive turbidity were filtered and remeasured. To determine the CO_2_ content, samples were brought to 25 °C in a water bath and then measured with a Haffmans Inpack 2000 CO_2_ calculator (Pentair Haffmans, Zürich, Switzerland). The turbidity of decarbonated samples at 25 °C was measured using a Hach^®^ 2100AN Turbidimeter (Hach^®^, Loveland, CO, USA). Hop acids were determined in the samples using high-performance liquid chromatography (HPLC) operated under conditions outlined in the European Brewing Congress (EBC) standard method 9.47 [20]. Iso-Humulones and Humulinones were quantified using the international calibration standards ICS-I4 and ICS-Hum1, while Humulones were quantified using the international calibration extract ICE4. Standards were purchased from Labor Veritas AG, Engimattstrasse 11, CH-8002 Zürich.

#### 2.4.2. Volatile Beer Analyses

To evaluate whether the samples used for both DA/CATA sensory and consumer analysis were dissimilar due to production differences (such as batch-to-batch variation), analysis of variance (ANOVA) was used to compare all the measured basic quality results (data not shown). Titratable acidity was the only quality specification that was significantly different between the samples used for these different evaluations. Therefore, it was assumed that the volatile analyses would also not be significantly different and were only performed on the samples used for the DA/CATA sensory. Duplicate measurements were run for each sample and then the average of these values was used for all the related multi-variate statistical modeling. Select hop aroma compounds (i.e., terpenes and oxygenated terpenes) were evaluated using HS-SPME-GC-MS/MS using published methodology [21,22]. Some other lower boiling point volatile compounds (i.e., esters, alcohols, and aldehydes) were measured using HS-GC-FID operated under conditions outlined in the EBC standard method 9.39 [20]. Dimethyl sulphide was measure using HS-GC-PFPD operated under conditions outlined by the Mitteleuropäische Brautechnische Analysenkommission (MEBACK^®^) 2.23.1.1 standard method [23]. The relative standard deviation for these measurements was < 10% for each analyte.

### 2.5. Statistical Analysis

Multi-factor analysis, three-way analysis of variance (ANOVA) (including the factors judge, sample, and replication as well as corresponding two-way interactions), multiple comparison analysis on data from all the trained panel replications and on all the consumers used for data analysis (Fisher’s least significant difference (LSD), *p* < 0.05), CATA analysis (i.e., Cochran’s Q test, *p* < 0.05), Pearson correlation analysis, principal component analysis (PCA), external preference mapping (PREFMAP), and graphical construction were carried out using XLSTAT 2020.1.1 (Addinsoft, New York, NY, USA). For cases in the ANOVA, where significant effects were observed for both the sample term and an interaction term including sample, a pseudo mixed model was performed using R-3.2.2 [24]. To determine the significance of the sample term a new F-value was calculated with the mean sum of squares from the significant interaction as the error term for the sample term (data not shown).

These tests and graphs were used to gauge the effectiveness of the judges in generating descriptive data, evaluate the significant differences in the aroma, taste, and mouthfeel profiles between the different NABs, and relate the consumer and trained beer, soda, and flavored sparkling water ratings to the chemical, sensory profiles, and hedonic results to determine which constituents and aromas, taste, and mouthfeels trigger Northern Californian consumers to perceive NABs as more beer like.

## 3. Results and Discussion

### 3.1. How Big Were the Differences in Beer Similarity between the Different Commercial NABs? How Similar Did the Consumer and Trained Panel Rate the Commercial NABs to “Beer”, “Soda”, and “Sparkling and/or Flavored Water”?

As seen by the increasing trend in beer similarity and the significant groupings (based on Fisher’s LSD test at *p* < 0.05), the consumer and trained panels observed clear differences in how similar the different commercial NABs were to beer, soda, and sparkling flavored water (Table 1). Out of all the products evaluated, the hoppy lager (L18) was rated as the most similar to beer by both the consumer and trained panels and on average the consumer panel rated L18 and IPA1 as being between moderately and very similar to beer. Except for the hop water (HW2) and the radler (R1), the consumer panel rated the rest of the products as being between slightly and moderately similar to beer. On average, both HW2 and R1 were perceived as being between moderately and slightly dissimilar to beer. In comparison, HW2 was perceived as being between moderately and very similar to sparkling flavored water by the consumer panel. While the consumers perceived R1 to be moderately similar to soda and slightly similar to sparkling flavored water. Ultimately, this is not that surprising because these products are much different than traditional NABs with the hop water containing only hops and no malted barley and the radler has a wheat base that has been blended with citrus juice.

**Figure 1:**
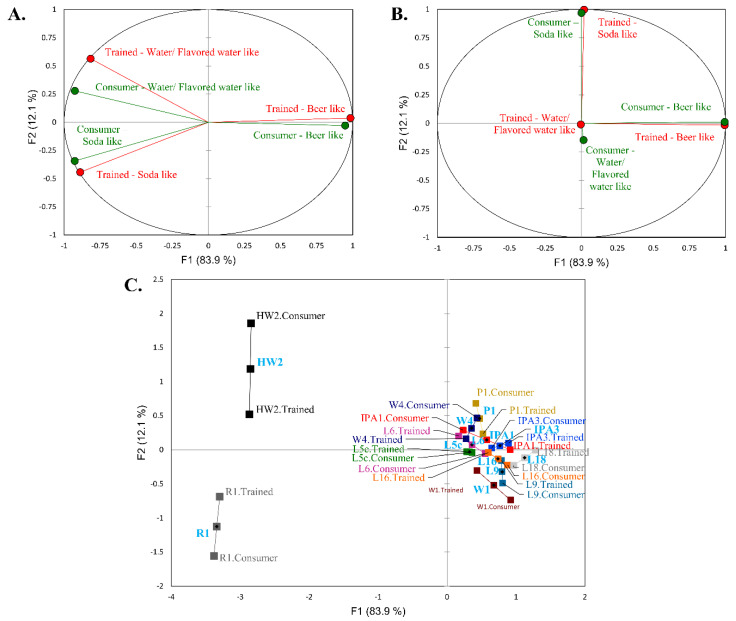
Multiple factor analysis was used to compare the least square means of the similarity ratings of beer, soda, and sparkling flavored water for each product tested in consumer analysis for both the trained and consumer panel. (**A**) Correlations between the similarity ratings of beer, soda, and sparkling flavored water ratings for the trained and consumer panels, (**B**) partial axes plot (i.e., the principal components of the individual PCAs for each panel), and (**C**) coordinates of the projected points for the 12 NABs for both the consumer and trained panel data.

Overall, similar to the hedonic data reported previously [8], there was generally good agreement between the beer, soda, and sparkling flavored water similarity ratings between the trained and consumer panels (Figure 1 and Table 2 and Table 3). Multiple factor analysis was applied to investigate the relationship between the beer, soda, and sparkling flavored water similarity ratings of the trained and consumer panels (Figure 1). The similarity ratings between the consumer and trained panels for the twelve samples were extremely associated (Figure 1A and Table 2 and Table 3). As can be seen, HW2 was rated to be the most similar to sparkling flavored water, R1 was rated to be the most similar to soda, while L18 was perceived to be the most beer like by both panels (Figure 1C). As defined previously [8], it is generally not good practice to collect opinion data from trained panelists. However, in this study, the trained panel was made up of judges who were regular beer consumers and not employees interconnected to the products. Further, they were not told about the similarity evaluations until after the DA testing sessions had finished. Given the current situation and the limitations of performing sensory amidst the COVID-19 pandemic, these results suggest that if the researcher is careful in the construction of the panel and always performs DA before any other additional survey, that a smaller trained team might be able to generate at least some actionable data that are descriptive of broader consumer trends. Nevertheless, ultimately one should always perform consumer analysis if they are able to.

### 3.2. The Aromas and Volatiles Linked with “Beer”, “Soda” and “Sparkling and/or Flavored Water” in Non-Alcoholic Beer

As described in Lafontaine, Senn, Dennenlöhr, Schubert, Knoke, Maxminer, Cantu, Rettberg and Heymann [8] Section 3.1. DA and CATA Results, ANOVA was used to confirm that statistically significant differences were observed for all of the DA terms after accounting for both panelist and replication effects. Similarly, the statistical significance of the replicated CATA terms was evaluated using (Cochran’s Q test *p* < 0.05). The aromas which were positively correlated with beer similarity for consumers were malty, cheerios, grape nuts, dried yeast, and skunk (Table 2). For the trained this was also true, except worty, banana, grassy, and stale were also associated with products perceived to be more similar to beer. However, this is not surprising because both “worty” and “stale” are very beer sensory specific descriptors and the trained panel was trained to identify these terms. The aromas negatively correlated with beer similarity were overall aroma intensity, citrus, lemon, orange, tropical, stone fruit, and cola. In general, for both the trained and consumer panels, these characteristics were positively correlated with products perceived to be more soda and sparkling flavored water like. Notably, these attributes along with floral aroma were also significantly positively correlated with the consumer aroma liking as well. This indicates that Northern Californian consumers were least satisfied with NABs that they perceived as more beer like, which had more malty, cheerio, grape nut, dried yeast, and skunk aromas typically associated with lager-style NAB (L18, etc.). The more preferred NABs, such as IPA1, HW2, and R1, had more fruity aromas driven by botanicals (such as dry hopping or citrus juice) and were perceived as more similar to soda and sparkling water by Northern Californian consumers.

No volatiles were positively correlated with beer likeness for the consumer panel (Table 4). However, for the trained panel, the volatiles significantly positively correlated with beer likeness were dimethylsulfide (DMS) and 3-methyl−1-butanol. Recently, Piornos et al. [25] determined both DMS (aroma quality—sweet corn) and 3-methyl-1-butanol (aroma quality—beer, malt) as important odor active compounds for a worty lager-style AFB. Therefore, the impact of these volatiles on beer flavor should be explored further. For example, dimethylsulfide has been identified as a key contributor of aroma in lager-beer styles that is derived from malted barley and yeast and there have been plenty of ways (i.e., either agriculturally or via the brewing process) which have been identified to control its concentration in beer [26]. Some hop varieties have also been shown to contain DMS [27] and the concentrations of DMS in hops are likely influenced by ripening [28]. Therefore, dry hopping with particular hop varieties at the right maturity could also be a novel way to extract DMS and impart beer-like flavor if this was a desired attribute. Similarly, investigating novel yeast strains or fermentation techniques to promote the formation of higher alcohols could lead to products to be perceived as more beer like. 

The volatiles negatively correlated to beer likeness for the consumer panel were heptanal, octanal, nonanal, decanal, trans, trans-2,4-decadienal, α-pinene, limonene, trans-linalool oxide, and α -terpineol. For the trained panel, geranyl acetate and caryophyllene were also negatively correlated to beer similarity. In general, these same volatiles were also positively correlated with soda like character. Most of these volatiles were also correlated to the aromas; citrusy, tropical, stone fruit, or floral, which most Northern Californian consumers were most satisfied with [8]. These compounds were also hypothesized to mask/suppress the aromas, cheerio, grape nut, and dried yeast, with which the consumers were least satisfied [8]. Prior research has shown that aliphatic straight-chain (*n*-) aldehydes such as octanal, nonanal, and decanal as well as terpenes, such as limonene, geranyl acetate, and α -terpineol are present in citrus oil and are important to the aroma of citrusy carbonated beverages [29]. The current results support this finding because the radler, R1, was one of the NABs that consumers were most satisfied with and it was a wheat NAB blended with lemon citrus juice and had high concentrations of these volatiles. Therefore, although the aroma profiles these products are being perceived by consumers as more “soda like” instead of “beer like”, one strategy to improve NAB aroma could be to add in fruit juices/oils.

Also notable was that benzaldehyde was positively correlated to the sparkling flavored water similarity ratings for both the trained and consumer panels (Table 4) and was the only volatile correlated with consumer aroma liking [8]. Linalool was also correlated to sparkling flavored water similarity but only for the trained panel. As mentioned previously [8], the dry-hopped hop water, HW2, had an aroma profile that most Northern Californian consumers were satisfied with which was characterized by citrusy, tropical, stone fruit aromas correlated to aldehydes (such as benzaldehyde), terpenes (such as linalool, nerol, citronellol, myrcene, and geraniol) and esters (such as 2-methyl butyl isobutyrate). In general, several studies have shown that the volatiles important for citrusy carbonated beverages are also important for the flavor of hops and hop aroma in hop-forward beer styles (such as IPA, pale ales, etc.) [30,31,32,33]. However, these results may indicate that the extraction of these compounds during dry-hopping might make these consumers perceive the aroma of these hop-forward aroma beer styles to be more like soda or sparkling flavored water. Although it is important to point out that the aroma quality imparted during dry-hopping depends on the hop varieties used, the amount of hops added, and the conditions under which dry hopping is performed [13]. For example, in this study hop aroma was also negatively correlated with soda-like character for the trained panelists (Table 2) and also positively correlated with herbal, black tea, grassy, and solvent aroma qualities (Supplementary Table S15 in [8]). Previously. these aroma qualities were hypothesized to arise in NABs which were produced with estimated dry hop rates above 800 g/L and it was suggested that these extreme levels may lead to the extraction of certain volatiles (i.e., hexanal, pentanal, etc.) which result in more of these herbal like aromas and suppress the more fruity aromas [8].

**Table 4:** Pearson correlation matrix of least square means for both the trained and consumer panel beer, soda, water similarity ratings, hedonic data, and volatile chemistry.

Variables	CBeer Like ^#^	TBeer Like *	CSoda Like ^#^	TSoda Like *	CWater Like ^#^	TWater Like *	COverall Liking ^#^	TOverall Liking *	CAroma Liking ^#^	TAroma Liking *	CTaste Liking ^#^	TTaste Liking *
2-methylpropanal	0.04	0.10	−0.04	−0.12	−0.17	−0.16	−0.28	−0.04	−0.44	0.00	−0.25	−0.04
ethyl nicotinate	0.32	0.20	−0.19	−0.13	−0.10	−0.21	0.04	0.02	−0.36	−0.01	0.06	−0.05
2-methylbutanal	−0.35	0.14	0.56	−0.03	0.06	−0.20	0.10	0.11	−0.19	0.17	0.15	0.07
3-methylbutanal	−0.21	0.26	0.53	0.02	−0.04	−0.26	0.18	**0.30**	−0.21	**0.31**	0.24	0.28
pentanal	−0.39	−0.08	**0.63**	−0.01	0.10	0.16	0.22	−0.01	−0.03	0.05	0.23	−0.05
hexanal	−0.56	−0.15	**0.77**	0.04	0.31	0.19	0.28	−0.05	0.13	0.02	0.33	−0.09
2-furfural	−0.45	−0.10	0.57	**0.40**	0.12	−0.23	0.00	0.19	−0.09	0.20	0.07	0.13
heptanal	**−0.58**	**−0.32**	**0.71**	**0.33**	0.23	0.22	0.15	−0.02	0.06	0.08	0.17	−0.07
methional	−0.10	0.14	0.28	0.10	−0.15	−0.28	0.04	0.26	−0.27	**0.33**	0.11	0.23
octanal	**−0.68**	**−0.41**	**0.85**	**0.54**	0.45	0.22	0.35	−0.01	0.23	0.06	0.39	−0.02
benzaldehyde	−0.57	−0.29	0.36	0.18	**0.77**	**0.47**	0.39	0.19	**0.67**	0.28	0.33	0.15
phenyl ethanal	0.03	0.29	0.33	−0.12	−0.22	−0.30	0.19	0.13	−0.22	0.22	0.25	0.08
nonanal	**−0.66**	**−0.48**	**0.81**	**0.64**	0.38	0.06	0.28	0.15	0.11	0.20	0.31	0.14
trans−2-nonenal	−0.05	0.14	0.06	0.03	−0.29	−0.20	−0.39	0.12	−0.41	0.16	−0.30	0.04
decanal	**−0.71**	**−0.44**	**0.86**	**0.57**	0.48	0.20	0.34	0.02	0.25	0.09	0.37	0.01
trans,trans-2,4-decadienal	**−0.59**	**−0.36**	**0.73**	**0.47**	0.29	0.13	0.13	0.01	0.02	0.08	0.19	−0.03
dimethyl sulfide	0.33	**0.32**	−0.41	−0.28	−0.50	**−0.31**	−0.37	−0.08	0.03	0.06	−0.44	−0.14
acetaldehyde	0.23	0.23	−0.06	−0.08	−0.09	−0.23	0.23	0.22	0.11	0.19	0.24	0.23
ethyl acetate	0.16	0.24	−0.07	−0.09	−0.07	−0.18	0.01	0.20	−0.10	0.10	0.06	0.21
propanol	−0.04	0.10	−0.38	−0.15	−0.28	−0.05	**−0.69**	**−0.35**	−0.35	−0.30	**−0.74**	**−0.35**
isobutanol	0.07	0.17	−0.08	−0.05	−0.27	−0.15	−0.21	0.14	−0.33	0.14	−0.23	0.14
isoamyl acetate	0.04	0.16	−0.21	−0.14	−0.19	−0.10	−0.38	0.00	−0.32	0.03	−0.37	0.03
2-methyl−1-butanol	0.08	0.19	−0.14	−0.05	−0.26	−0.14	−0.30	0.17	−0.40	0.12	−0.29	0.19
3-methyl−1-butanol	0.20	**0.33**	−0.12	−0.12	−0.25	−0.27	−0.10	0.30	−0.25	0.20	−0.08	**0.32**
phenylethyl acetate	0.24	0.26	−0.07	−0.05	−0.25	−0.25	−0.01	**0.31**	−0.28	0.22	0.07	**0.33**
phenyl ethanol	0.15	0.09	−0.03	−0.03	−0.02	−0.10	0.05	0.02	−0.30	−0.06	0.10	0.01
α-pinene	**−0.68**	−0.19	**0.85**	0.23	0.45	0.22	0.35	−0.08	0.24	0.01	0.39	−0.10
ß-pinene		−0.18		0.22		0.22		−0.08		0.01		−0.10
myrcene	−0.46	−0.20	0.23	0.14	0.14	0.21	−0.22	−0.25	0.13	−0.04	−0.28	−0.20
2-methylbutylisobutyrate	−0.22	−0.02	−0.15	−0.21	0.16	0.24	−0.22	**−0.35**	0.41	−0.15	−0.34	**−0.35**
limonene	**−0.68**	**−0.48**	**0.85**	**0.64**	0.45	0.20	0.35	0.03	0.24	0.07	0.39	0.02
cis-linalool oxide	−0.33	−0.21	0.10	0.15	0.08	0.05	−0.26	−0.24	0.15	−0.14	−0.31	−0.28
trans-linalool oxide	**−0.58**	**−0.46**	0.47	**0.61**	0.28	−0.07	−0.06	0.17	0.24	0.19	−0.09	0.15
linalool	−0.38	−0.14	−0.07	−0.04	0.19	**0.33**	−0.40	**−0.36**	0.26	−0.15	−0.49	**−0.39**
α-terpineol	**−0.69**	**−0.54**	**0.86**	**0.76**	0.46	−0.05	0.35	0.25	0.25	0.24	0.39	0.24
citronellol	0.00	0.04	−0.33	−0.12	−0.25	0.08	−0.55	**−0.43**	0.05	−0.18	**−0.64**	**−0.43**
nerol	−0.22	−0.14	−0.24	0.00	0.01	0.30	−0.50	**−0.34**	0.14	−0.08	**−0.60**	**−0.35**
geraniol	−0.14	−0.04	−0.31	−0.17	−0.09	0.21	**−0.58**	−0.29	0.00	−0.09	**−0.67**	−0.29
geranyl acetate	−0.30	**−0.35**	0.21	**0.43**	0.13	0.14	−0.03	−0.08	0.47	0.05	−0.11	−0.10
caryophyllene	−0.25	**−0.39**	0.38	**0.53**	0.09	0.07	0.18	0.04	0.41	0.15	0.13	0.03
humulene	0.21	0.03	−0.18	−0.07	−0.27	0.00	−0.09	−0.13	0.21	−0.01	−0.17	−0.13
caryophyllene oxide	−0.15	−0.22	0.13	0.23	0.48	0.28	0.36	0.08	0.31	0.15	0.31	0.08

^#^ Based on only the 12 samples evaluated during consumer analysis (in blue). * Based on the 44 samples evaluated during trained panel analysis. Values in bold are different from 0, with a significance level alpha = 0.05 and positive and negative correlations highlighted by green and red respectively.

Notably, skunk (“light-struck) aroma has been related to the direct and indirect irradiation of iso-α-acids and the subsequent formation of 3-methylbut-2-ene-1-thiol (3-MBT) [34]. This is notable because L18 was packaged in a green bottle and was also rated as the highest in skunk quality. Further, one of the NABs was tested in two package types, L5c (can) and L5b (green bottle), and although not statistically significant, the sample packaged in the green bottle was rated as being more similar to beer as well as higher in skunk quality. Given that hops, iso-α-acids, and the formation of 3-MBT are largely unique to beer production it is not surprising that this characteristic would invoke beer similarity. Even though 3-MBT was not measured in this study, the formation of 3-MBT has been shown to be impacted through the use of reduced hop acids and/or light-filtering packaging (i.e. cans and/or brown bottles) [35]. Therefore, this suggests that the choice of packaging also influences the flavor characteristics influencing beer similarity.

**Figure 2:**
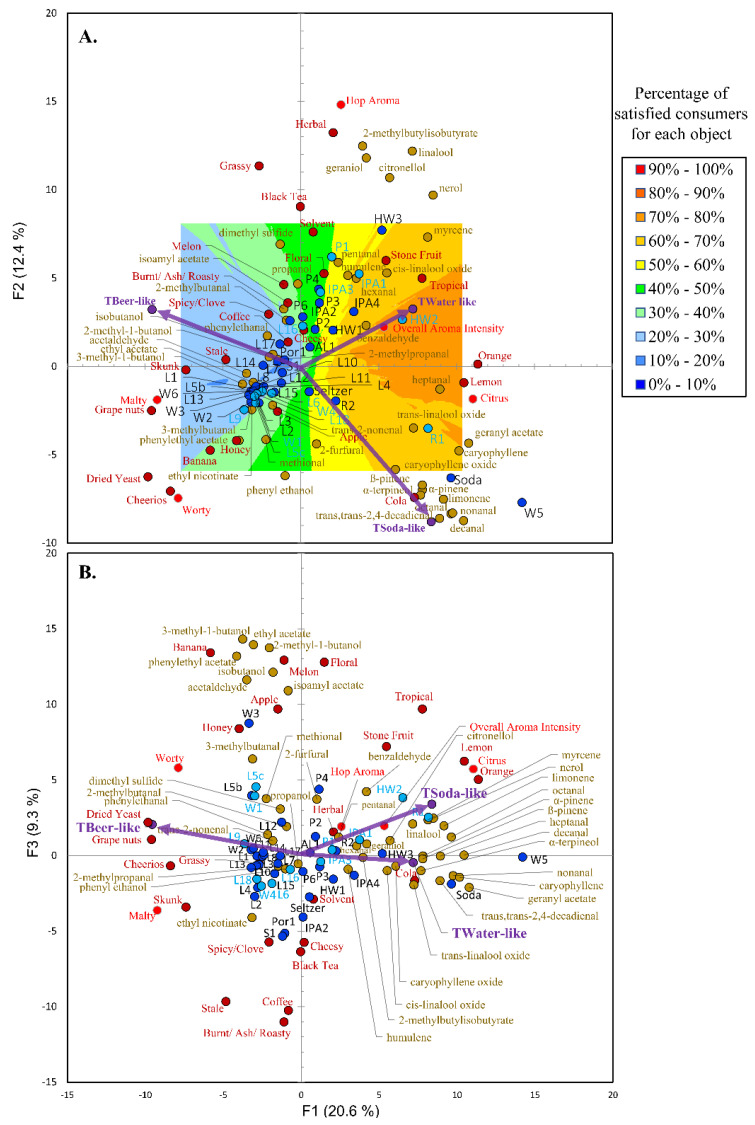
Principle component analysis biplots of the beer-like, soda-like, water/flavored water-like ratings (purple circles/arrows, Table 1) for the trained panel along with the statistically different aroma attributes (Light red circles (DA terms) and dark red circles (CATA terms)) amongst the different products (light- and dark-blue circles, Table 1) and volatiles (light brown circles). The samples highlighted by the dark blue circles were evaluated only by the trained panel, whereas the samples highlighted by the twelve light blue circles were also evaluated by consumer analysis. PC1 and PC2 (**A**) described 33.0% of the variation in the data, while PC1 and PC3 (**B**) accounted for an additional 9.3%. External preference mapping (using F-ratios to find the best vector model, *p* < 0.05, contour plot threshold % = 100) was performed as described in Section 3.2. The consumers were least satisfied with the products in the dark blue region, whereas consumers were more satisfied with the products in the dark red/orange region.

**Figure 3:**
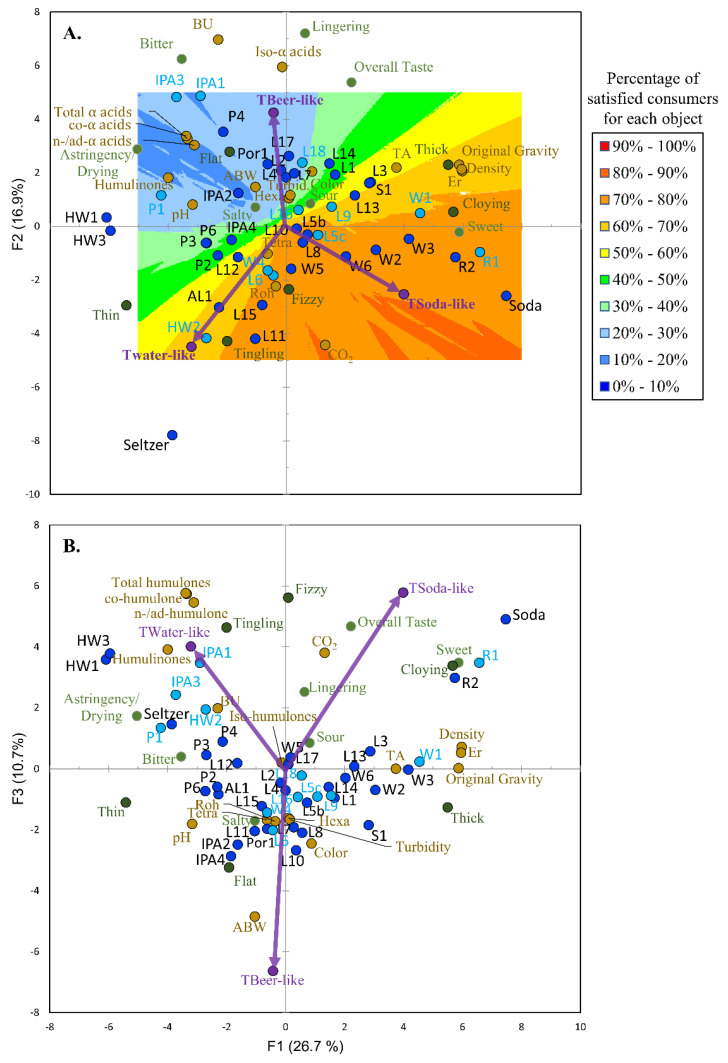
Principle component analysis biplots of the beer-like, soda-like, water/flavored water-like ratings (purple circles/arrows, Table 1) for the trained panel along with the statistically different taste/mouthfeel attributes (light green circles (DA terms) and dark green circles (CATA terms)) amongst the different products (light- and dark-blue circles, Table 1) and non-volatile factors (light brown circles). The samples highlighted by the dark blue circles were evaluated only by the trained panel, whereas the samples highlighted by the twelve light blue circles were also evaluated by consumer analysis. PC1 and PC2 (**A**) described 43.6% of the variation in the data, while PC1 and PC3 (**B**) accounted for an additional 10.7%. External preference mapping (using F-ratios to find the best vector model, *p* < 0.05, contour plot threshold % = 100) was performed as described in Section 3.2. The consumers were least satisfied with the products in the dark blue region, whereas consumers were more satisfied with the products in the dark red/orange region.

A similar procedure to what was outlined previously [8] was used to visualize the relationships between the similarity ratings with the aroma and volatile chemistry as well as with the taste/mouthfeel non-volatile chemistry. In brief, PCA was run on the correlation matrix of the aroma attributes, volatile compounds, and beer, soda, and sparkling flavored water similarity ratings of the trained panel (Figure 2), as well as on the correlation matrix of just the taste/mouthfeels, the non-volatile factors, and the beer, soda, and sparkling flavored water similarity ratings of the trained panel (Figure 3). The PCA factor scores from only the 12 samples evaluated via consumer analysis were then taken and related to aroma (Figure 2) and taste/mouthfeel (Figure 3) liking scores of the consumers to generate vector models using external preference mapping (PREFMAP). This resulted in contour plot which could be used to visualize the percentage of satisfied consumers (i.e., satisfaction for the set 12 samples was classified as the percentage of consumers giving a liking rating above the mean liking rating). The dark blue regions represent a low percentage of satisfied consumers. In comparison, the dark-red regions represent a high percentage of satisfied consumers. Again, the 12 products highlighted by light-blue circles were evaluated by consumer analysis.

The PCA, made using the correlation matrix of the trained panel similarity ratings, the DA/CATA aroma data, and the volatile chemistry data along with the external preference mapping contour plot, can be used to visualize the discussion in this section (Figure 2). For example, beer similarity is a key feature that described PC1 and as one moves from right to left in the plots, (Figure 2A, B), the NABs were perceived to be more beer like and were characterized by the aromas (i.e., skunk, malty, stale, grape nuts, dried yeast, etc.) associated with beer described earlier in this section. The external preference map contour plots also show that these consumers were least satisfied (i.e., the blue region) with products that were more similar to beer. Also, notable is that the NABs with aromas perceived to more similar to soda were in the bottom right-hand quadrant, while the NABs perceived to be more similar to sparkling flavored water were in the top right-hand quadrant. The soda control data support this finding because it sits in the bottom right-hand quadrant. Again, based on the contour plots, it can be seen that the consumers were generally more satisfied (i.e., the red/orange regions) with the products (e.g., HW1, R1, and IPA1) which had aromas (i.e., stone fruit, tropical, citrus, lemon, orange, cola, etc.) characterized by increased terpenes, esters, aliphatic straight-chain aldehydes, as well as benzaldehyde and which were perceived as more similar to soda and sparkling flavored water. Not surprisingly, the seltzer control sits almost on the origin because it was not perceived to have any aroma.

### 3.3. The Non-Volatiles Associated with the Tastes and Mouthfeels Linked with “Beer”, “Soda” and “Sparkling and/or Flavored Water” in Non-Alcoholic Beer

Only one taste was close to being suggestively positively correlated with beer similarity for consumers and this was bitterness (*p*-value = 0.175, Table 3). For the trained panel, bitterness was significantly correlated with beer similarity (*p*-value = 0.008, Table 3). The only taste which was significantly negatively correlated with beer similarity for consumers was sourness. This was also the case for the trained panel, but tingling was also significantly negatively correlated with beer similarity. In comparison, the tastes and mouthfeels positively correlated with soda similarity for consumers were sweet, cloying, and sour, while bitterness was negatively correlated. Similar relationships were again observed for the trained panel. For consumers, overall aroma and taste liking were also significantly positively correlated with soda and sparkling flavored water.

As mentioned in the introduction, although different styles are defined by varying levels of hop bitterness, the main compounds (i.e., iso-α-acids) leading to hop bitterness in beer are unique to beer production [10,11,13]. Interestingly, unlike the volatiles associated with dry hopping, the concentration of iso-α-acids, associated with kettle hopping, were significantly positively correlated with beer similarity and significantly negatively correlated with soda and sparkling flavored water similarity. This finding supports the uniqueness of these compounds to beer and NAB production and indicates that the addition of iso-α-acids to NAB makes them more beer like. It is important to point out that consumers were least satisfied with NABs with high bitterness intensity which was moderately correlated with high concentrations of iso-α-acids [8]. Consequently, brewers must also consider the role that residual extract, as well as artificial sweeteners, have in suppressing beer bitterness to develop NABs with taste profiles that are more palatable to consumers. Notably, residual extract was significantly positively correlated to soda like character for the trained panel (Table 3) suggesting that the NABs (e.g., R1) with higher residual extracts were perceived as more soda like. This is not surprising because the residual extract in soda is also extremely high (Table 1). However, prior research found that Dutch and Portuguese consumers considered NAB as a healthier adult alternative beverage to both alcoholic beverages (i.e., beer) and soft drinks because of their reduced sugar content [36]. Therefore, the caloric content of the product must also be an important consideration in the development of NABs and potentially natural artificial sweeteners could be a way to achieve NABs that have a perceived sweetness but a lower caloric content.

Distinctly, while sourness is not correlated to beer similarity, it was correlated with soda and sparkling water like characteristics as well as also positively correlated to overall, aroma, and taste liking. This is highlighted analytically because pH was significantly positively correlated to beer similarity and negativity correlated to soda-like character, indicating that NABs with higher pH were more beer like/less soda like and those with lower pH were perceived as less beer like/more soda like. Further evident from the trained panel is that titratable acidity was positively correlated to soda-like character. It should be noted that this does not mean that there are not sour beer styles. However, what this may suggest is that due to the prevalence and dominance of lager beer styles on the beer market, the average consumer in Northern Californian might still not be privy to beer styles with these types of flavor characteristics and/or these consumers only consider beer styles with lager flavor characteristics as beer like. As an example, based on these findings even though Berliner Weisse is a historical beer style that has been produced since the 16th century, it might not be a beer style which these consumers consider beer given its sour and fruity aroma characteristics [37]. While no commercial Berliner Weisse style NABs were tested in this study, it could be an untapped style to probe for NAB development because it is a style with a fairly low ABV (~3%) and has flavor characteristics which were generally preferred by the Northern Californian consumers in this study. However, one would have to be careful in marketing this adult alternative beverage correctly and these results might caution breweries from using the term “beer” to market this type of product, as it is clear this term is being associated with more lager beer style flavor characteristics for Northern Californian consumers. This also supports prior research [36], that suggested that NAB should be marketed better and that direct conceptual comparisons with beer (although the authors do not specify it is assumed they are referring to lager beer styles), particularly regarding flavor, should be avoided to mitigate consumer disappointment.

Further, observable for the trained panel was that CO_2_ concentration and tingling character were negatively correlated with beer likeness. Suggesting that NABs with lower CO_2_ concentrations were less tingling and more beer like. As shown previously [8], the average CO_2_ concentration in the NABs was ~5 g/L, with the wheat styles containing some of the highest ammounts~6 g/L and the commercial soda/seltzer controls containing ~7 g/L. Furthermore, dissolved gas not only imparts a distinctive taste and mouthfeel to beverages, but also acts as a preservative because it prevents bacterial, yeast, and mold growth [38].

Another interesting observation is that alcohol by weight (ABW) was positively correlated to beer similarity for the trained panel. This is not surprising and although this is not a possible strategy to make alcohol free beer, one technique employed by commercial brewers to make the flavor profile of NAB more palatable is to blend full-strength beer either to a dealcoholized product or to a product produced with an arrested/modified fermentation so that the ABW is just under the legal permissible limit [39]. One way to explore this further would be to analyze the glycerol concertation of the products because glycerol significantly increases during primary and secondary fermentation as it is related to the multiplication of biomass and/or the enhancement of ethanol content in fully fermented beers [40]. Therefore, blended NABs would presumably contain higher levels of glycerol.

The PCA made using the correlation matrix of the trained panel similarity ratings, the DA and CATA taste/mouthfeel data, and the non-volatile chemistry data along with the external preference mapping contour plot can be used to visualize the discussion in this section (Figure 3). For example, beer similarity was a key feature that generally described the top left-hand corner of the plot of PC1 and PC2 (Figure 3A) and as one moves towards this quadrant, the NABs (i.e., P4, IPA3, IPA1, etc.) were perceived as more beer like and were characterized by taste/mouthfeels (i.e., bitterness, lingering, etc.) associated to increasing concentrations of iso-α-acids. As with the aromas driving beer similarity, the external preference map contour plots also show that the Northern Californian consumers were least satisfied (i.e., the blue region) with products that had taste/mouthfeel profiles perceived to be more similar to beer. PC3 was also characterized by beer similarity and both ABW and pH increase, while CO_2_ decreases as you move from top to bottom in the plot of PC1 and PC3 (Figure 3B). In comparison, soda similarity was related to PC1 (bottom right-hand corner), and as one moves from left to right in the plot (Figure 3A) the NABs (i.e., R1, R2, W1, etc.) which were generally perceived as more soda like were more satisfying to consumers (i.e., the red/orange region) and had taste profiles characterized as sweeter, cloying, and sour which was associated with their higher residual extracts (Er) and lower pH. Again, the data for the soda control support this finding because it also sits in the bottom right-hand quadrant. Similarly, the NABs (i.e., HW2, Al1, HW3, etc.) perceived as more like sparkling flavored water in taste/mouthfeel were more in the bottom left-hand corner of the plot (Figure 3A) and characterized by thin and tingling mouthfeels associated with high CO_2_ concentrations. Again, these products had taste/mouthfeel profiles which were more satisfying (i.e., yellow/orange region) than the beer-like profiles and the seltzer control supports this finding because it sits in the bottom left-hand corner of the plot.

## 4. Conclusions

Overall, these results suggest that Northern Californian consumers were generally not satisfied with NABs styles (mostly lager styles) perceived as more beer like in aroma (i.e., skunk, malty, stale, grape nuts, dried yeast, etc.) and beer like in taste and mouthfeel (i.e., bitterness—associated with iso-α-acids). A clean and crisp character is what makes up a good lager beer but this is hard to achieve in a NAB. Increases in dimethyl sulfide, as well as higher alcohols (3-methyl-1-butanol), were also associated with more beer-like NABs. Similarly, NABs with higher ABWs, pH, iso-α-acids, and lower CO_2_ concentrations were perceived to be more beer like.

However, consumers were more satisfied with NABs perceived as more like soda and sparkling flavored water. Generally, these NABs (HW2, R1, IPA1, etc.) were perceived to have more fruity aromas (i.e., stone fruit, tropical, citrus, lemon, orange, cola, etc.—associated with increased terpenes, esters, aliphatic straight-chain aldehydes, as well as benzaldehyde). The more soda-like NABs (i.e., R1, etc.) had taste/mouthfeels which were perceived to be more sour and sweeter which were associated with higher residual extracts and pH, while the more sparkling flavored water-like NABs (i.e., HW2, etc.) had taste/mouthfeels perceived to be thin and tingling associated with higher CO_2_ concentrations. These results are thought-provoking because as mentioned in the discussion there are already historical and commercial examples of beer styles (i.e., IPAs, Berliner Weisse, etc.) with the types of flavor characteristics Northern Californian consumers are more satisfied with although they might consider them more soda like. Further, these soda/sparkling flavored water-like aroma and taste/mouthfeel characteristics could be imparted to NABs by natural and historical production brewing techniques (such as kettle souring, dry hopping, etc.) and with typical beer ingredients (such as hops).

These results also suggest that the average Northern Californian consumer considers the flavor profiles more associated with lager styles to be more beer like. Given the extreme prevalence and dominance of lager beer on the beer market, this is not a surprising result. However, the variety of beer styles that are already commercially and historically exist could be an untapped development tool in the search for new NABs that would have more preferable flavor profiles to Northern Californian consumers. Though because North American consumers from California have a preconceived notion of what “beer flavor” is, similar to prior research [36], one would have to be careful in the marketing of these new products so that they not only mitigate consumer disappointment but draw in new consumers. However, more work is needed to determine whether these findings would be reflective of the preference and opinions of consumers in other markets (i.e., countries). Ultimately, future beer and NAB drinkers will be consumes who have not only been familiarized to lager beer but have been introduced to a variety of beer styles through craft beer. In Germany, for example, shifting trends in the market can already be seen because newer hop-forward beers and NABs (i.e., IPAs) are becoming more prevalent and are taking market share from the more flagship NAB brands (i.e., lager brands) which have historically made up a lion share of the beer and NAB market.

Overall, these results are instructive for beverage and food companies which are trying to develop novel substitute and/or alternative products (i.e. NAB, fake meats, dealcoholized wine, etc.) because they suggest that understanding the most salient drivers of flavor as well as the consumer preferences/opinions of the products being reimagined are both critical considerations for developing and marketing new products which can best meet consumer expectations.

## Figures and Tables

**Table 1:** Non-alcoholic beer, soda, and seltzer basic non-volatile quality specifications as well as how similar the consumer panel and trained panel rated the products to beer, soda, and water.

Product Code	Brewery	Brand Style	Er (%w/w)	ABW (%w/w)	pH	BU	CBeer Like ^#^	TBeer Like *	CSoda Like ^#^	TSoda Like *	Cwater Like ^#^	TWater Like *
Soda	11	Soda	10.76	0	2.47	3.2		0.4 j		8.9 a		0.6 hi
Seltzer	23	Seltzer	0.09	0	5.83	0.03		0.6 ij		0.9 fgh		6.7 a
R1	22	Wheat with lemon	9.03	0.11	3.43	7	3.5 f	0.8 ij	7.0 a	7.9 ab	6.1 b	3.2 cd
R2	26	Lager with lemon	8.18	0.01	3.74	10.4		1.2 ij		7.0 b		2.8 cde
HW1	14	Hop water	0.21	0.01	5.27	26.7		1.8 hij		1.4 efgh		5.8 ab
HW2	19	Hop water	0.27	0.02	3.68	8.6	3.9 f	2.2 ghi	4.9 b	3.4 cd	7.5 a	6.9 a
W5	27	Wheat with orange	3.85	0.12	4.21	10.4		3.0 gh		4.4 c		4.2 bc
AL1	25	Kolsch	1	0.19	4.36	9.1		3.7 fg		2.0 defg		4.2 c
HW3	16	Hop/tea water	0.49	0	5.11	18.1		3.8 fg		1.1 fgh		4.2 bc
L12	24	Lager	0.46	0	3.58	16.1		5.0 ef		2.7 de		2.6 cdefg
S1	27	Coffee Stout	7.75	0.28	4.51	12.3		5.0 ef		1.2 efgh		2.1 defghi
IPA2	25	Red IPA	1.76	0.33	4.13	20.7		5.1 ef		0.4 gh		2.3 defgh
L15	4	Lager	3.63	0.29	4.38	9.2		5.3 def		0.9 fgh		2.7 cdef
Por1	25	Porter	2.68	0.24	4.27	19.9		5.3 def		0.2 h		1.5 defghi
IPA4	25	Hazy IPA	1.63	0.19	4.61	12.4		5.5 cde		1.3 efgh		2.3 cdefgh
L11	12	Lager	3.55	0.32	3.87	8.4		5.5 cde		1.2 efgh		2.3 cdefgh
P4	5	Golden	4.34	0.05	4.44	41.7		5.5 bcde		0.6 gh		1.8 defghi
L7	27	Amber	4.49	0.21	4.46	22.4		5.7 abcde		0.8 fgh		0.9 efghi
P6	21	Blonde	1.76	0.33	4.18	17.8		5.7 abcde		1.0 fgh		1.8 defghi
W4	27	Wheat	3.92	0.22	4.2	10.8	6.5 cd	5.8 abcde	2.9 def	0.3 h	4.2 cd	1.9 defghi
IPA1	5	IPA	5.2	0.34	4.31	45.4	7.2 b	5.8 abcde	2.7 ef	1.0 fgh	3.5 ef	2.0 defghi
L3	7	Pilsner	8.92	0	5	20.1		5.8 abcde		2.4 def		1.9 defghi
L2	7	Pilsner	6.21	0.06	4.52	26.1		5.9 abcde		0.9 fgh		1.1 efghi
W6	13	Wheat	6.64	0.2	4.43	14.8		5.9 abcde		1.2 efgh		1.0 efghi
P3	21	Pale Ale	1.76	0.33	4.14	19.7		6.0 abcde		0.7 gh		1.9 defghi
P1	8	Pale Ale	1.03	0.32	4.74	28.4	5.8 e	6.1 abcde	2.2 g	0.3 h	3.5 ef	2.3 cdefgh
IPA3	21	IPA	1.73	0.29	4.05	56.9	6.3 d	6.1 abcde	2.2 g	0.6 gh	3.1 f	1.2 efghi
L8	2	Amber	5.24	0.26	4.3	10.6		6.1 abcde		1.9 defg		1.3 defghi
L17	10	Hoppy lager	6.78	0.23	4.71	33.5		6.1 abcde		0.8 fgh		1.5 defghi
L6	2	Golden	4.14	0.39	4.38	10.2	6.8 bc	6.2 abcde	3.1 cde	1 fgh	4.6 c	1.3 defghi
L5c	15	Lager	5.31	0.03	4.43	13.1	6.8 bcd	6.2 abcde	3.3 cd	1.4 efgh	4.1 cd	1.6 defghi
L1	1	Pilsner	6.83	0.04	4.54	21.9		6.2 abcde		0.9 fgh		1.3 defghi
L13	3	Lager	6.79	0.03	4.51	22		6.2 abcde		1.2 efgh		0.7 hi
W2	6	Wheat	6.76	0.29	4.25	12.5		6.3 abcde		1.9 defg		0.5 hi
L9	20	Amber	6.45	0.04	4.21	20.8	6.8 bc	6.4 abcde	2.9 def	1.1 fgh	3.2 f	0.5 hi
L16	9	Hoppy lager	6.36	0.36	4.52	24.6	6.9 bc	6.4 abcde	3.0 cdef	0.6 gh	3.7 de	0.7 ghi
W3	18	Wheat	7.51	0.27	4.49	10.9		6.4 abcde		2.0 defg		1.2 efghi
L14	10	Lager	6.7	0.3	4.78	25.2		6.6 abcde		1.7 efgh		0.8 fghi
P2	17	Pale Ale	3.3	0.25	4.73	15.4		6.8 abcd		1.0 fgh		2.1 defghi
W1	18	Wheat	8.64	0	4.31	16.6	6.5 cd	7.0 abc	3.4 c	1.6 efgh	3.3 ef	0.3 i
L4	26	Pilsner	6.16	0.33	4.68	20.4		7.0 abc		0.3 h		0.6 hi
L5b	15	Lager	5.28	0.02	4.4	14		7.0 abc		0.8 fgh		0.6 hi
L10	20	Lager	4.95	0.58	4.21	14		7.2 ab		1.3 efgh		0.9 fghi
L18	26	Hoppy lager	6.26	0.26	4.71	26.2	7.7 a	7.2 a	2.5 fg	1.0 fgh	3.3 ef	0.9 fghi
						**LSD**	0.46	1.65	0.46	1.57	0.54	1.91

^#^ Least square means based on the results of the consumer panel on 12 samples evaluated during consumer analysis (in blue). * Least square means based on the results of the trained panel on only the 44 samples evaluated during trained panel analysis. The columns are sorted by the trained beer likeness ratings and colored low to high similarity. Letters represent significant groupings based on Fisher’s least significant difference (LSD) test at *p* < 0.05. The ratings for the panels were as follows; 1. extremely dissimilar, 2. very dissimilar, 3. moderately dissimilar, 4. slightly dissimilar, 5. neither similar nor dissimilar, 6. slightly similar, 7. moderately similar, 8. very similar, and 9. extremely similar. Abbreviations: real extract (Er), bitterness unit (BU), and alcohol by weight (ABW).

**Table 2:** Pearson correlation matrix of least square means for both the trained and consumer panel beer, soda, water similarity ratings, hedonic data, and aroma attributes.

Variables	CBeer Like ^#^	TBeer Like *	CSoda Like ^#^	TSoda Like *	CWater Like ^#^	TWater Like *	COverall Liking ^#^	TOverall Liking *	CAroma Liking ^#^	TAroma Liking *	CTaste Liking ^#^	TTaste Liking *
CBeer like	**1.00**											
Tbeer like	**0.94**	**1.00**										
CSoda like	**−0.84**	**−0.48**	**1.00**									
Tsoda like	**−0.81**	**−0.71**	**0.97**	**1.00**								
CWater like	**−0.84**	**−0.47**	**0.79**	**0.39**	**1.00**							
TWater like	**−0.78**	**−0.71**	0.56	0.17	**0.90**	**1.00**						
COverall liking	−0.29	−0.26	**0.66**	**0.31**	**0.65**	0.23	**1.00**					
TOverall liking	0.00	0.15	0.35	0.29	0.30	**−0.33**	**0.74**	**1.00**				
CAroma liking	−0.47	**−0.34**	0.55	0.27	**0.74**	**0.42**	**0.75**	0.17	**1.00**			
TAroma liking	−0.05	0.25	0.31	0.27	0.31	**−0.39**	**0.70**	**0.87**	0.47	**1.00**		
CTaste liking	−0.25	−0.25	**0.66**	**0.32**	**0.60**	0.19	**0.99**	**0.43**	**0.66**	**0.37**	**1.00**	
TTaste liking	−0.01	0.15	0.31	0.27	0.27	**−0.32**	**0.66**	**0.96**	0.17	**0.82**	**0.68**	**1.00**
Overall aroma intensity	**−0.60**	−0.03	0.47	**0.31**	0.41	−0.17	0.08	−0.10	0.56	0.19	0.04	−0.17
Citrus	**−0.83**	**−0.57**	**0.89**	**0.65**	**0.75**	**0.46**	0.53	0.00	**0.70**	0.14	0.50	−0.04
Hop aroma	0.24	0.20	−0.49	**−0.36**	−0.33	0.12	−0.41	**−0.41**	0.14	−0.16	−0.51	**−0.38**
Worty	0.40	**0.49**	−0.10	−0.09	−0.44	**−0.59**	−0.06	**0.47**	**−0.58**	**0.40**	0.07	**0.44**
Malty	**0.72**	**0.68**	**−0.65**	**−0.43**	**−0.79**	**−0.67**	−0.51	0.01	**−0.84**	0.06	−0.42	−0.04
Cheerios	**0.68**	**0.50**	−0.40	−0.17	−0.47	**−0.50**	0.02	**0.41**	−0.52	**0.32**	0.12	**0.39**
Grape nuts	**0.61**	**0.68**	**−0.58**	**−0.44**	**−0.68**	**−0.62**	−0.51	0.29	**−0.84**	0.24	−0.42	0.29
Dried yeast	**0.72**	**0.66**	−0.47	**−0.33**	−0.49	**−0.62**	−0.03	**0.49**	−0.53	**0.38**	0.07	**0.51**
Banana	0.23	**0.37**	−0.06	−0.07	−0.25	**−0.33**	−0.03	**0.45**	−0.34	**0.30**	0.02	**0.46**
Spicy/Clove	0.36	0.17	−0.14	−0.18	−0.45	−0.26	0.05	−0.27	−0.22	−0.15	0.08	−0.30
Lemon	**−0.75**	**−0.52**	**0.77**	**0.59**	**0.78**	**0.43**	0.53	0.09	**0.74**	0.25	0.49	0.05
Orange	**−0.83**	**−0.58**	**0.86**	**0.66**	**0.73**	**0.44**	0.48	−0.02	**0.70**	0.12	0.44	−0.05
Tropical	**−0.77**	**−0.34**	**0.63**	**0.32**	**0.87**	**0.52**	0.49	0.06	**0.84**	0.24	0.41	0.06
Stone fruit	**−0.66**	−0.27	0.41	0.14	**0.77**	**0.54**	0.29	−0.02	**0.68**	0.15	0.23	−0.06
Melon	−0.36	0.13	0.18	−0.12	0.51	0.19	0.24	0.20	**0.59**	**0.33**	0.19	0.17
Apple	−0.32	0.09	0.32	0.17	0.40	0.01	0.20	0.26	0.07	0.28	0.22	0.27
Floral	−0.50	0.05	0.50	0.09	0.36	0.07	0.29	0.15	**0.61**	0.30	0.20	0.15
Herbal	0.16	0.17	−0.37	−0.25	−0.33	−0.01	−0.39	−0.27	0.03	−0.05	−0.51	−0.29
Black tea	0.53	0.07	−0.54	**−0.30**	−0.47	0.02	−0.16	**−0.34**	−0.13	−0.20	−0.21	**−0.36**
Grassy	0.51	**0.49**	**−0.71**	**−0.49**	**−0.67**	−0.25	**−0.66**	−0.29	−0.43	−0.12	**−0.71**	−0.22
Cheesy	0.27	0.08	−0.37	−0.23	−0.32	0.03	−0.47	**−0.35**	−0.47	**−0.34**	−0.46	**−0.35**
Honey	0.03	0.19	0.07	0.12	−0.05	**−0.31**	0.01	**0.37**	−0.02	**0.37**	0.11	**0.35**
Stale	0.28	**0.32**	−0.38	**−0.46**	−0.21	−0.17	−0.29	−0.26	−0.50	−0.27	−0.30	−0.20
Solvent	0.08	0.00	−0.20	−0.18	−0.28	0.13	−0.34	**−0.48**	−0.15	**−0.46**	−0.35	**−0.45**
Skunk	**0.76**	**0.63**	**−0.64**	**−0.45**	−0.57	**−0.44**	−0.23	0.18	−0.56	0.17	−0.16	0.19
Coffee	−0.09	0.01	−0.29	−0.17	−0.16	−0.03	**−0.58**	**−0.48**	−0.39	**−0.41**	**−0.61**	**−0.50**
Burnt/Ash/Roasty	0.18	0.09	**−0.58**	−0.25	−0.50	−0.10	**−0.83**	**−0.52**	−0.48	**−0.44**	**−0.89**	**−0.55**
Cola	**−0.62**	**−0.54**	**0.83**	**0.77**	0.40	−0.06	0.35	0.22	0.19	0.21	0.41	0.21

^#^ Based on only the 12 samples evaluated during consumer analysis (in blue). * Based on the 44 samples evaluated during trained panel analysis. Values in bold are different from 0, with a significance level alpha = 0.05 and positive and negative correlations highlighted by green and red respectively. The characteristics in light red were evaluated by descriptive analysis, while the characteristics in dark red were determined by replicated CATA.

**Table 3:** Pearson correlation matrix of least square means for both the trained and consumer panel beer, soda, water similarity ratings, hedonic data, taste and mouthfeel attributes, and non-volatile chemistry.

Variables	CBeer Like ^#^	TBeer Like *	CSoda Like ^#^	TSoda Like *	CWater Like ^#^	TWater Like *	COverall Liking ^#^	TOverall Liking *	CAroma Liking ^#^	TAroma Liking *	CTaste Liking ^#^	TTaste Liking *
CBeer like	**1.00**											
TBeer like	**0.94**	**1.00**										
CSoda like	**−0.84**	**−0.48**	**1.00**									
TSoda like	**−0.81**	**−0.71**	**0.97**	**1.00**								
CWater like	**−0.84**	**−0.47**	**0.79**	**0.3** **9**	**1.00**							
TWater like	**−0.78**	**−0.71**	**0.56**	0.17	**0.90**	**1.00**						
COverall liking	−0.29	−0.26	**0.66**	**0.31**	**0.65**	0.23	**1.00**					
TOverall liking	0.00	0.15	0.35	0.29	0.30	**−0.33**	**0.74**	**1.00**				
CAroma liking	**−0.47**	**−0.34**	**0.55**	0.27	**0.74**	**0.42**	**0.75**	0.17	**1.00**			
TAroma liking	−0.05	0.25	0.31	0.27	0.31	**−0.3** **9**	**0.70**	**0.87**	0.47	**1.00**		
CTaste liking	−0.25	−0.25	**0.66**	**0.32**	**0.60**	0.19	**0.99**	**0.43**	**0.66**	**0.37**	**1.00**	
TTaste liking	−0.01	0.15	0.31	0.27	0.27	**−0.32**	**0.66**	**0.96**	0.17	**0.82**	**0.68**	**1.00**
Overall taste intensity	−0.30	−0.09	0.20	**0.40**	−0.11	**−0.30**	−0.34	−0.15	0.04	0.08	−0.34	−0.24
Astringency/Drying	0.05	0.18	−0.48	**−0.49**	−0.14	0.21	**−0.56**	**−0.47**	0.09	−0.27	**−0.64**	**−0.50**
Sour	**−0.75**	**−0.33**	**0.76**	**0.36**	**0.69**	0.16	0.39	−0.27	0.44	−0.08	0.37	**−0.31**
Salty	−0.07	−0.05	−0.07	−0.07	0.06	0.14	−0.26	**−0.40**	−0.20	**−0.36**	−0.25	**−0.41**
Lingering	0.14	0.24	−0.25	−0.02	−0.47	**−0.42**	**−0.54**	−0.28	−0.13	−0.02	**−0.54**	**−0.38**
Bitter	0.42	**0.39**	**−0.67**	**−0.54**	**−0.60**	−0.18	**−0.71**	**−0.62**	−0.23	**−0.38**	**−0.74**	**−0.66**
Sweet	−0.47	−0.26	**0.79**	**0.78**	0.31	**−0.32**	0.48	**0.57**	0.14	**0.53**	0.55	**0.54**
Thin	−0.09	−0.09	−0.22	**−0.36**	0.33	**0.52**	−0.06	**−0.39**	0.30	**−0.33**	−0.15	**−0.38**
Thick	0.18	0.22	0.16	0.24	−0.31	**−0.56**	0.12	**0.41**	−0.39	**0.33**	0.22	**0.41**
Flat	0.40	0.07	−0.15	−0.27	−0.04	0.08	0.35	**−0.33**	0.08	−0.23	0.41	**−0.35**
Fizzy	−0.16	−0.29	0.18	0.25	−0.11	0.14	−0.15	0.11	0.16	−0.03	−0.17	0.11
Cloying	−0.36	−0.28	**0.67**	**0.69**	0.15	−0.26	0.29	**0.38**	−0.03	**0.30**	0.37	**0.34**
Tingling	−0.32	**−0.37**	−0.07	0.11	0.30	**0.41**	−0.21	−0.02	0.17	−0.10	−0.32	0.02
Er	0.15	0.12	0.34	**0.42**	−0.21	**−0.61**	0.39	**0.54**	−0.11	**0.49**	0.46	**0.50**
ABW	0.40	**0.48**	−0.47	**−0.37**	−0.34	**−0.33**	−0.25	−0.11	−0.09	−0.01	−0.31	−0.12
pH	**0.82**	0.25	**−0.83**	**−0.65**	−0.73	0.21	−0.40	−0.30	−0.51	**−0.42**	−0.39	−0.24
TA	−0.36	0.04	**0.68**	**0.32**	0.22	**−0.33**	0.43	0.19	0.17	0.22	0.45	0.15
BU	0.40	**0.39**	**−0.58**	**−0.41**	**−0.59**	−0.27	**−0.63**	**−0.37**	−0.17	−0.16	**−0.66**	**−0.44**
Iso-α-acids	**0.57**	**0.51**	**−0.59**	**−0.30**	**−0.66**	**−0.49**	**−0.51**	−0.01	−0.25	0.08	**−0.49**	−0.09
Humulinones	−0.15	−0.02	−0.29	−0.22	−0.03	0.27	**−0.45**	**−0.39**	0.15	−0.21	**−0.56**	**−0.38**
Total α-acids	0.31	−0.10	−0.41	−0.17	−0.37	0.27	−0.33	**−0.33**	0.12	−0.19	−0.38	**−0.34**
co- α-acids	0.28	−0.07	−0.40	−0.18	−0.36	0.23	−0.34	**−0.30**	0.12	−0.13	−0.40	**−0.32**
*n*-/ad- α-acids	0.34	−0.13	−0.40	−0.15	−0.37	0.29	−0.29	**−0.34**	0.14	−0.24	−0.34	**−0.33**
Roh		0.02		−0.04		0.03		0.05		0.05		0.03
Tetra	0.14	0.19	−0.10	−0.09	−0.23	−0.05	−0.11	0.12	−0.24	0.19	−0.04	0.08
Hexa		0.04		−0.07		−0.11		−0.02		0.03		−0.03
CO_2_	−0.31	**−0.40**	0.16	0.29	0.26	0.20	−0.04	0.25	−0.04	0.01	−0.08	0.29
Color	0.26	−0.01	−0.26	−0.07	−0.54	−0.13	−0.31	**−0.39**	−0.28	**−0.32**	−0.36	**−0.43**
Turbidity	−0.40	−0.04	0.57	−0.01	0.11	0.01	0.13	−0.21	0.12	−0.17	0.13	−0.19

^#^ Based on only the 12 samples evaluated during consumer analysis (in blue). * Based on the 44 samples evaluated during trained panel analysis. Values in bold are different from 0, with a significance level alpha = 0.05 and positive and negative correlations highlighted by green and red respectively. The characteristics in light green were evaluated by descriptive analysis, while the characteristics in dark green were determined by replicated CATA.
